# A Dual-Functional Zr-Ion Crosslinked PVA-Alginate Hydrogel with Embedded ZrMgFe-LDH for Enhanced Phosphate Recovery

**DOI:** 10.3390/gels12070570

**Published:** 2026-06-28

**Authors:** Fengqin Tang, Runwen Xiong, Shiqi Zou, Xiaomei Ma, Beibei Sun, Hui Bai, Libing Hu, Peng Chen

**Affiliations:** 1 School of Chemistry and Chemical Engineering, Tarim University, Alar 843300, China; tfqbuct@163.com (F.T.); 19326680857@163.com (R.X.); 18146483695@163.com (S.Z.); 13150338120@163.com (X.M.); beibeisun0410@163.com (B.S.); hlbin101@126.com (L.H.); 2 State Key Laboratory of Chemical Resource Engineering, Beijing University of Chemical Technology, Beijing 100029, China; 3 Analysis and Testing Center, Tarim University, Alar 843300, China

**Keywords:** phosphate removal, ZrMgFe-LDHs, alginate hydrogel microspheres, water purification, slow-release fertilizer

## Abstract

Excess phosphate in aquatic environments can trigger eutrophication and pose risks to ecosystem integrity and public health, even though phosphate is indispensable for plant growth. Herein, we report the fabrication of Zr-LDHs-PS hydrogel microspheres by in situ cross-linking zirconium–magnesium–iron layered double hydroxides (ZrMgFe-LDHs) with Polyvinyl alcohol (PVA) and sodium alginate (SA). The resulting bead-type adsorbent was designed to enable efficient phosphate capture from water while facilitating subsequent, controlled phosphate release. Benefiting from the cross-linking granulation strategy, the microspheres mitigate typical limitations of powdered adsorbents, including compaction, aggregation, and poor separability. General characterization (SEM, FT-IR, XPS, XRD, BET, TG, and zeta potential) elucidated the microstructure and surface chemical composition. The Zr-LDHs-PS microspheres exhibited a maximum experimental adsorption capacity of 51.313 mg/g. Kinetics data were best fitted by the pseudo-second-order model, and adsorption isotherms were subjected to the Freundlich model, pointing to heterogeneous, multilayer adsorption. Importantly, high phosphate selectivity was preserved despite the coexistence of competing anions (Cl^−^, NO_3_^−^, and CO_3_^2−^). After adsorption, the spent beads released phosphate gradually in water, highlighting their potential for dual functionality. Collectively, these results demonstrate that Zr-LDHs-PS hydrogel microspheres are promising candidates for extraction-based phosphate removal and resource recovery, with prospects for repurposing slow-release phosphate fertilizers to support sustainable plant nutrition.

## 1. Introduction

Eutrophication of water bodies poses a global environmental challenge, with excessive accumulation of phosphate being one of its key drivers [1,2]. Beyond pollution control, phosphorus is a strategic and non-renewable resource essential for agriculture; therefore, simultaneous phosphate removal and recovery (resource reutilization) have become an increasingly important objective in water treatment [3]. To address these needs, efficient and practical technologies for phosphate capture from wastewater are critically required. Currently, Conventional phosphate removal in wastewater treatment mainly relies on enhanced biological phosphorus removal and chemical precipitation [4]. However, biological processes are often limited by insufficient carbon sources, and chemical precipitation is highly pH-dependent. In contrast, adsorption is operationally simple and energy-efficient, and is particularly effective for polishing low-concentration phosphate [5].

To remove phosphate from water bodies, various adsorbents have been extensively explored. These include calcite [6], activated carbon and biochar [7], montmorillonite [8], metal oxides [9], Metal–Organic Framework [10], porous silica [11] and layered double hydroxides (LDHs) [12]. Among them, metal ions such as Fe, Mg, and Zr have demonstrated strong affinity and immobilization capability toward phosphate [13]. Based on this property, composites formed by intercalating zirconium ions into LDH structures have emerged as efficient phosphate adsorbents. The removal rate approaches 99% for 25 mg/L phosphorus solutions within 120 min [14]. LDHs possess a host-guest architecture, composed of brucite-like layers bearing a positive charge, with intercalated anions and water molecules counterbalancing this charge [15]. Among them, Mg-Fe LDHs are particularly attractive for environmental remediation because of their green composition, strong phosphate affinity, low cost, and facile synthesis. Notably, Mg and Fe are essential plant nutrients, which may enable agronomic co-benefits if spent sorbents are repurposed after water treatment [16,17].

However, conventional Mg-Fe LDHs often show limited phosphate uptake and poor selectivity in complex water matrices. To overcome these drawbacks, Zr^4+^ doping has been demonstrated as an effective strategy. Zr^4+^ can provide highly active sites through strong specific coordination with phosphate (PO_4_^3−^) [18]. While its high charge density strengthens electrostatic attraction between LDH layers and phosphate, thereby enhancing both adsorption capacity and selectivity [19]. For instance, Zhang et al. [20] reported a Zr-doped MgFe-Zr LDHs, which achieved a phosphate removal rate of 96.4% under optimized conditions. Its adsorption capacity was 1.86 times that of the undoped material.

Although ZrMgFe-LDH nano-sized powders exhibit superior performance for phosphate adsorption, they face significant challenges in practical engineering applications, similar to many powdered adsorbents. Difficulties in solid–liquid separation due to fine particles, the risk of secondary pollution from material loss, and incompatibility with continuous-flow processes severely limit their large-scale use. To address this separation challenge, several strategies have been developed. As an example, Drenkova-Tuhtan et al. [21] immobilized Mg-Fe-Zr LDH on Fe_3_O_4_@SiO_2_ microspheres, producing a composite that allows for facile magnetic separation. This value falls short of that for pure LDH powder by approximately two orders of magnitude. Overall, existing immobilization methods often improve separation at the cost of reduced adsorption performance. Therefore, there is imperative to develop low-cost and efficient technical approaches that can effectively cement the separability of LDHs while maintaining their high phosphate adsorption capacity.

Hydrogel-based immobilization techniques have garnered significant interest owing to their excellent hydrophilicity and structural tunability [22]. PVA, a polyhydroxy polymer, can be crosslinked chemically or physically to form hydrogels. In wastewater treatment, crosslinked PVA is highly regarded for its safety, elasticity, and mechanical durability. On the other hand, SA can bind with multivalent metal ions (M^n+^, *n* > 2) by exchanging Na^+^ and H^+^, enabling the formation of hydrogels with improved stability, durability, and good biodegradability [23]. By blending PVA and SA, a polyvinyl alcohol–sodium alginate (PS) hydrogel with a three-dimensional porous network can be synthesized. Its large specific surface area provides space for hosting LDH powders, offering a potential solution to the separation challenge of LDH powders [24]. However, directly adding inert organic crosslinkers without adsorption function often reduces the adsorption capacity of composites by 50–54% [25]. Introducing reactive metal ions into the PS network, serving both as crosslinkers and modifiers, could further enhance phosphate adsorption performance. Accordingly, we propose Zr-LDHs-PS beads in which Zr^4+^ plays a dual role—modifying LDHs and crosslinking the PVA/alginate network—an underexplored strategy for constructing recoverable phosphate sorbents.

In this work, Zr-LDHs-PS hydrogel microspheres were prepared via gelation-assisted in situ cross-linking using PVA and SA as the matrix and ZrMgFe-LDHs as the active phase. For comparison, M-LDHs-PS beads (M = Ce, La, Zr) were synthesized by cross-linking different metal-ion solutions, and key parameters (e.g., Zr^4+^ concentration and LDH loading) were optimized. Phosphate adsorption was assessed using kinetics and isotherm experiments, together with the impact of pH and competing anions, and the data were analyzed with the pseudo-second-order and Freundlich models. XPS and surface-charge analyses (zeta potential and pHpzc) provided insights into the underlying adsorption mechanism. Finally, controlled phosphate release from the spent beads was assessed to explore their potential for post-treatment reuse as slow-release phosphate sources.

## 2. Results and Discussion

### 2.1. Effect of Formulation Conditions on Adsorption Performance

#### 2.1.1. Zr^4+^ Doping Content in LDHs

**Figure 1:**
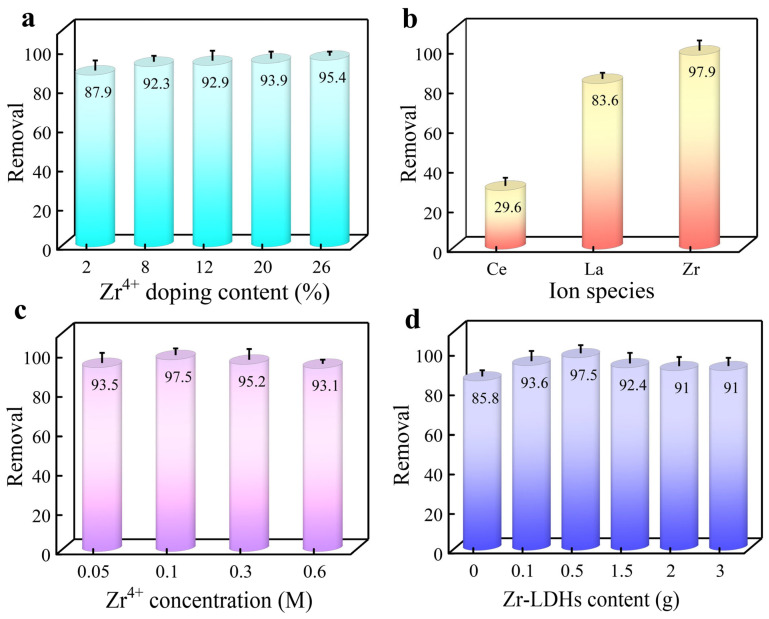
(**a**) Phosphate adsorption by Zr-LDHs with different Zr^4+^ doping contents; (**b**–**d**) effects of cross-linking conditions on phosphate adsorption during hydrogel synthesis: (**b**) metal ion species, (**c**) Zr^4+^ concentration, and (**d**) Zr-LDHs mass.

Zr^4+^ was introduced during the synthesis of LDHs to modify its phosphate adsorption capability [26]. As presented in Figure 1a, as the Zr^4+^ doping ratio, expressed as R = Zr^4+^/(Zr^4+^ + Fe^3+^), increased from 2% to 26%, the phosphate removal rate rose from 87.9% to 95.4%. This trend indicates that the adsorption performance is enhanced with increasing Zr^4+^ content. It is noteworthy that at R = 8%, the phosphate removal efficiency already exceeded 90% even at an initial concentration of 50 mg/L. Although further increasing the Zr^4+^ ratio could still improve adsorption, based on an optimal balance between performance and cost, R = 8% was chosen as the condition for all follow-on experiments.

#### 2.1.2. Ion Species, Zr^4+^ Concentrations and Zr-LDH Content

In the preparation of Zr-LDHs-PS hydrogel adsorbents, the phosphate adsorption performance is influenced by the species and concentration of metal ions in the crosslinking solution, as well as the amount of Zr-LDHs added [27]. As shown in Figure 1b, the phosphate removal efficiencies varied with different metal ions in the crosslinking solution. The Zr^4+^-based crosslinking solution exhibited the best performance, achieving a removal rate 3.3 times that of Ce^3+^ and 1.2 times that of La^3+^. Therefore, the adsorptive capability of Zr-LDHs-PS for treatment necessitates further investigation. Furthermore, based on the gelation profile of the Zr-LDHs-PS hydrogel (Figure 1c), hydrogel beads were formed using varying concentrations of Zr^4+^ (0.05 M–0.3 M). Increasing the concentration of Zr^4+^ (0.05 M–0.1 M) led to an enhancement in phosphate removal efficiency from 93.5% to 97.5%, confirming the active role of Zr within the bead matrix. However, a continued rise in Zr^4+^ concentration to 0.6 M led to a decrease in removal efficiency to 93.1%. This reduction implies that the adsorption capacity for phosphate did not continue to improve and suggests that the cross-linking between SA and Zr^4+^ approached saturation. Since the SA content remained constant in the beads, progressively raising the Zr^4+^ concentration merely increased the concentration of metal ions without contributing to further adsorption performance.

Additionally, it is necessary to analyze the implications of Zr-LDHs content (0–3 g) in the adsorbent on adsorption performance (Figure 1d). The results demonstrate that increasing the Zr-LDHs content from 0 to 0.5 g significantly enhances phosphate adsorption capacity. At 0.5 g of Zr-LDHs, the removal rate reaches 97.5%. However, further increasing the content (0.5 g–3 g) leads to a decline in phosphate adsorption from 97.5% to 91%. This decrease is attributed to reduced stability and loss of adsorption sites, which hinder phosphate uptake. Similar conclusions have been corroborated in other studies [28].

### 2.2. Adsorption Kinetics

The study commenced with an investigation of the phosphate adsorption kinetics on the Zr-LDHs-PS hydrogel [29]. To evaluate the adsorption rate and explore the mechanism, the kinetics data were modeled with both pseudo-first-order and pseudo-second-order equations, as presented below:

(1) $$q_{t}=q_{e}(1-{exp}^{{-k}_{1}t})$$

(2) $$q_{t}=\frac{{qe}^{2}k_{2}t}{1+q_{e}k_{2}t}$$

In which q*_e_* and q*_t_* (mg/g) denote phosphate adsorption capacities of equilibrium and time t (min), respectively. k_1_ and k_2_ represent the rate constants for pseudo-first-order and the pseudo-second-order models.

**Figure 2:**
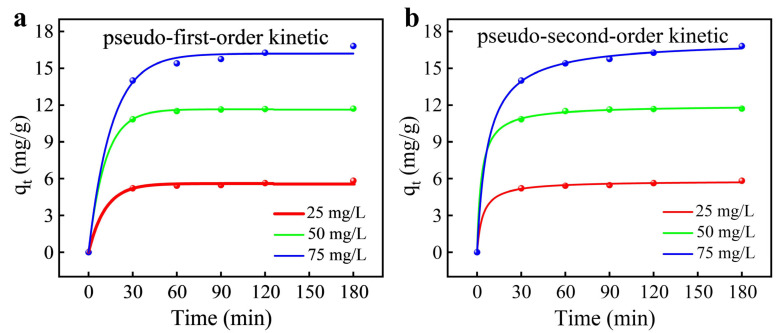
Kinetics modeling of phosphate adsorption on Zr-LDHs-PS hydrogels at initial concentrations of 25, 50, and 75 mg/L: (**a**) pseudo-first-order and (**b**) pseudo-second-order models.

A series of experiments were conducted to evaluate the mass transfer properties of the Zr-LDHs-PS hydrogel. The adsorption behavior over time was researched using phosphate solutions at 25 mg/L, 50 mg/L, and 75 mg/L, as shown in Figure 2 and Figure S1. The process exhibited high initial efficiency, attaining >85% removal within 30 min (89.6%, 93.2%, and 86.4% for 25, 50, and 75 mg/L, respectively). As time progressed, the adsorption rate gradually decreased. The corresponding maximum adsorption capacities reached 5.8 mg/g, 12.0 mg/g, and 17.3 mg/g, respectively. The 3D porous network of hydrogels facilitates rapid phosphate adsorption and promotes its diffusion and binding to the active sites within Zr-LDHs-PS. With the progressive occupation of these active sites, the reaction rate gradually decreases until adsorption equilibrium is eventually reached. A comparison of the fitting to both models is presented in Figure 2a,b. The kinetics data were best described by the pseudo-second-order model, which achieved high regression coefficients (R^2^ > 0.99) at all concentrations studied (Table 1). This points to chemical adsorption as a controlling factor alongside diffusion in the phosphate adsorption mechanism onto the Zr-LDHs-PS hydrogel. Ligand exchange, leading to the formation of strong chemical bonds, accounts for this observed behavior. Agreement with the pseudo-second-order kinetics model confirms chemical adsorption as the rate-controlling process. The adsorption mechanism was further elucidated by XPS analysis.

### 2.3. Adsorption Isotherms

**Figure 3:**
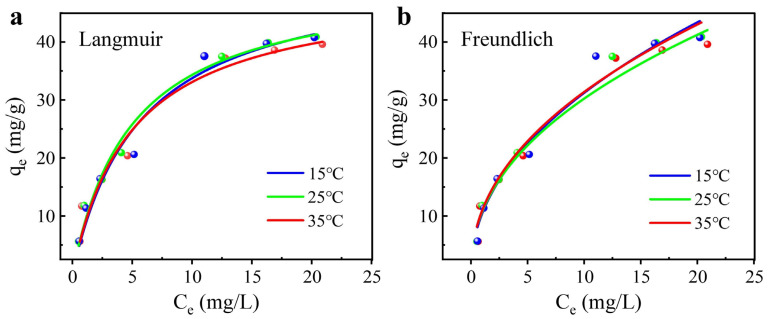
Adsorption isotherms of phosphate by the Zr-LDHs-PS hydrogel were simulated using the (**a**) Langmuir and (**b**) Freundlich models.

To determine the nature of adsorption and the maximum adsorption capacity, Figure 3 presents the adsorption isotherms of phosphate on the Zr-LDHs-PS hydrogel at different temperatures (15 °C, 25 °C, and 35 °C). The phosphate adsorption isotherms were described using the Langmuir and Freundlich equations:

(3) $$q_{e}=\frac{q_{m}K_{L}C_{e}}{1+K_{L}C_{e}}$$

(4) qe=KfCe1n

where q*_m_* and q*_e_* represent the adsorption capacities (mg/g) of equilibrium and maximum, respectively. C*_e_* (mg/L) denotes the equilibrium concentration. For the Freundlich model, K*_f_* and n characterize adsorption capacity and intensity. while K*_L_* (L/mg) in the Langmuir model corresponds to the adsorption energy.

Figure 3a and Figure S2 show that phosphate adsorption increases with rising temperature. An experimental q_m_ of 53.217 mg/g was achieved by the Zr-LDHs-PS hydrogel at 35 °C. However, the equilibrium constant K*_L_* follows the trend K*_L_* (15 °C) < K*_L_* (25 °C) > K*_L_* (35 °C), with a maximum value of 0.203 at 25 °C. This indicates that the affinity between the Zr-LDHs-PS hydrogel and phosphate is highest at 25 °C. Typically, the Langmuir model fits monolayer adsorption on homogeneous adsorbents, and the Freundlich model fits multilayer adsorption on heterogeneous ones, respectively [30]. Based on the R^2^ values of the two models listed in Table 2, the Freundlich model better describes the phosphate adsorption approach on the Zr-LDHs-PS hydrogel. This suggests that the adsorption tends to occur on a relatively heterogeneous surface, likely involving multilayer adsorption. It also indirectly indicates that the adsorption process is diffusion-controlled, with faster mass transfer and diffusion at higher temperatures. In the Freundlich model, a lower 1/n value indicates enhanced adsorption capability of the given material. In this study, the 1/n value for the Zr-LDHs-PS hydrogel is 0.455, clearly demonstrating the advantageous phosphate adsorption capability of the Zr LDHs PS hydrogel.

It should be noted that ZrMgFe-LDHs possess a typical layered structure, which provides additional interlayer spaces for anion exchange. The better fit to the Freundlich model compared to the Langmuir model suggests heterogeneous and multilayer adsorption, consistent with the involvement of both external surface adsorption and interlayer intercalation. Although direct evidence of interlayer spacing expansion was not quantitatively analyzed, the XPS shifts and kinetics results support the occurrence of ligand exchange and inner-sphere complexation, which are likely to occur both on the external surface and at the edge of interlayer galleries. The pseudo-second-order kinetics further confirm chemisorption as the rate-controlling step, which is typical for LDH-based adsorbents where interlayer anion exchange contributes significantly to the overall uptake capacity.

The Zr-LDHs-PS hydrogel demonstrates exceptional phosphate adsorption performance, outperforming many materials listed in Table 3. This advantage stems from its synergistic features: a 3D porous network that promotes diffusion and abundant LDH-layer -OH groups that drive the adsorption process through anion exchange and complexation.

### 2.4. Effects of Environmental Conditions on the Adsorption of Zr-LDHs-PS

**Figure 4:**
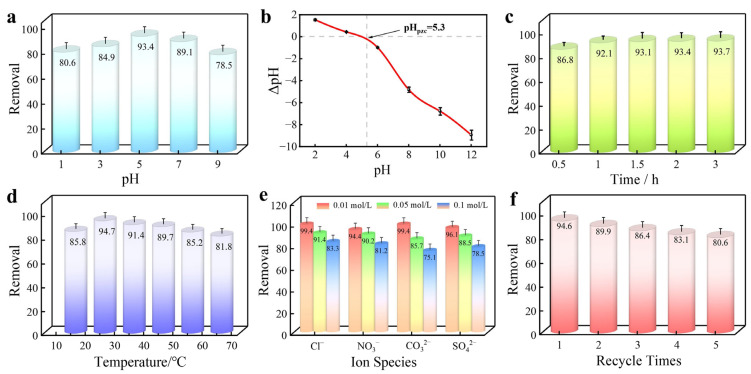
Influence of pH (**a**), temperature (**d**) and coexisting anions (**e**) on phosphate adsorption by Zr-LDHs-PS; (**b**) the point of zero charge for Zr-LDHs-PS; the adsorption result (**c**) and cycling performance (**f**) of Zr-LDHs-PS for phosphate adsorption at 25 °C.

To appraise the incidence of environmental conditions on the phosphate adsorption property of the Zr-LDHs-PS hydrogel, this study investigated the effects of solution pH, temperature, adsorption time, coexisting ions, and adsorbent reusability. A series of batch adsorption experiments conducted at initial pH values ranging from 1 to 9 (adjusted using 0.1 M HCl or NaOH), with other parameters held constant (C_0_ = 50 mg/L, T = 25 °C, contact time = 2 h). As shown in Figure 4a, the removal efficiency at different pH values (phosphate concentration: 50 mg/L) exhibited a clear pH-dependence. The pHpzc of Zr-LDHs-PS was determined to be 5.3 (Figure 4b). The highest removal efficiency (93.4%) was achieved at pH = 5, which is slightly below the pHpzc. Under this condition, the material surface carried a weak positive charge, while phosphate predominantly existed as H_2_PO_4_^−^. The resulting electrostatic attraction facilitated phosphate adsorption. At pH = 3, the surface positive charge was stronger, but phosphate was mainly present as neutral H_3_PO_4_ molecules. Electrostatic interaction was weakened, and extreme acidity may have caused partial dissolution or aggregation of the material. Consequently, the removal efficiency decreased moderately to 84.9%. At pH = 1, the same reasons (dominance of H_3_PO_4_ and reduced material stability) led to a further drop to 80.6%. When the solution pH exceeded the pHpzc (5.3), the material surface became negatively charged, causing electrostatic repulsion with phosphate anions. Thus, the removal efficiency decreased to 89.1% (pH = 7) and 78.5% (pH = 9). The structural stability of the Zr-LDHs-PS hydrogel was evaluated by visual inspection after 24 h immersion in deionized water at pH 3, 5, 7, and 9 (Figure S4). The beads maintained their spherical shape and structural integrity without visible disintegration or solution turbidity across all tested pH conditions, confirming the stability of the hydrogel network in the pH range of 3–9. These results collectively confirm that electrostatic attraction is the dominant mechanism for phosphate adsorption.

Zeta potential analysis provided crucial evidence (Figure S3). Before adsorption, the material surface showed a strong positive charge (+14.33 mV), facilitating the initial electrostatic accumulation of phosphate. After adsorption, the potential dropped sharply to nearly neutral (−0.06 mV). This confirms that phosphate anions were firmly bound to the surface, neutralizing the positive charge sites and pointing towards a strong specific chemisorption process [35]. This conclusion is further supported by the kinetics model (Figure 2b). In summary, the adsorption of phosphate onto Zr-LDHs-PS is a surface-charge-assisted process, with ligand-exchange chemisorption at its core. This mechanism ensures highly efficient phosphate removal across a broad pH range.

Figure 4c shows the phosphate removal by Zr-LDHs-PS from a 50 mg/L solution over time. The removal efficiency exceeded 90% after 1 h and reached a maximum of 93.7% after 3 h. The effect of temperature on the adsorption of phosphate (50 mg/L) by Zr-LDHs-PS was further investigated (Figure 4d). As shown, the removal rate increased with temperature from 15 °C, peaking at 94.7% at 25 °C. Further temperature rise led to a gradual decline in removal, reaching 77.3% at 70 °C. Obviously, the chemical adsorption process of Zr-LDHs-PS hydrogel is an exothermic reaction. According to Le Chatelier’s principle, higher temperatures will inhibit this adsorption [36]. If applied for environmental phosphate removal, Zr-LDHs-PS can be considered reliable under common outdoor temperature conditions, given its high removal efficiency at ambient temperature.

Figure 4e shows the impact of coexisting anions on phosphate adsorption process of by Zr-LDHs-PS. Phosphate adsorption was reduced with elevated concentrations of competing ions. The extent of this inhibition is closely related to the valence state of the anions. Monovalent anions (Cl^−^ and NO_3_^−^) had a minor impact, whereas divalent anions (CO_3_^2−^ and SO_4_^2−^) caused significant interference. This occurs because anions with higher valence states possess greater affinity for the positively charged layers. Consequently, they compete more effectively for adsorption sites, leading to a pronounced reduction in phosphate uptake capacity [37]. The phosphate-loaded Zr-LDHs-PS was regenerated using 1 mol/L NaOH at 25 °C for 1.5 h, followed by freeze-drying. As shown in Figure 4f, after five consecutive adsorption–desorption cycles, the phosphate removal efficiency gradually decreased but remained above 80%. The release of metal ions (Mg^2+^, Fe^3+^, and Zr^4+^) from the Zr-LDHs-PS hydrogel was quantified by ICP-OES (Figure S5). At the optimal pH of 5, metal leaching was negligible (<0.4 mg/L for all metals), confirming the chemical stability of the hydrogel network. Even under more aggressive conditions (pH 3–9), total metal release remained below 1.0 mg/L, demonstrating that the dual-crosslinked structure effectively prevents the loss of active components and minimizes the risk of secondary pollution.

### 2.5. Characterization

#### 2.5.1. SEM-EDS

**Figure 5:**
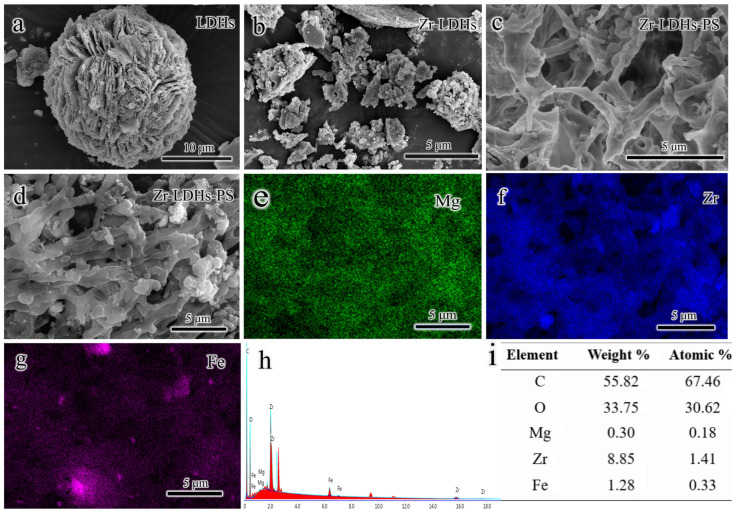
SEM images of the (**a**) LDHs, (**b**) Zr-LDHs, (**c**,**d**) Zr-LDHs-PS hydrogel; (**e**–**g**) elemental mapping showing the distribution of Mg, Fe, and Zr, respectively; (**h**,**i**) depict the spectra acquired from the PS-La-LDH hydrogel before adsorption.

The surface morphology and internal structure of adsorbents significantly influence their application performance. SEM images of the Zr-LDHs-PS hydrogel were collected, as shown in Figure 5. The SEM image of Zr-LDHs is presented in Figure 5b. Compared with the undoped LDHs shown in Figure 5a, which exhibit a flower-like morphology, the Zr-LDH powder consists of stacked irregular lamellar structures [38]. Such stacking results in a higher population of retained CO_3_^2−^ interlayer anions, which in turn boosts the anion exchange capacity. The layered structure also facilitates the entry of larger phosphate molecules into the interlayers, thereby improving material performance [39]. Figure 5c,d show the Zr-LDHs-PS hydrogel doped with Zr-LDHs. A well-defined porous architecture is evident inside the beads, arising from the irregular growth of ice crystals during the freeze-drying process. These pores provide sufficient specific surface area and abundant sites for phosphorus adsorption. The skeletal structure and surface micropores facilitate phosphate entry into the beads and hinder their easy release, leading to more thorough pollutant removal. The Zr-LDHs powder is attached to the Zr-LDHs-PS hydrogel framework and encapsulated within the hydrogel walls, which may result in lower adsorption efficiency compared to the powder form [40,41]. EDX analysis (Figure 5e–i) indicates that the material mainly contains the elements Fe, Mg, Zr, C, and O, which are uniformly dispersed.

#### 2.5.2. FT-IR

**Figure 6:**
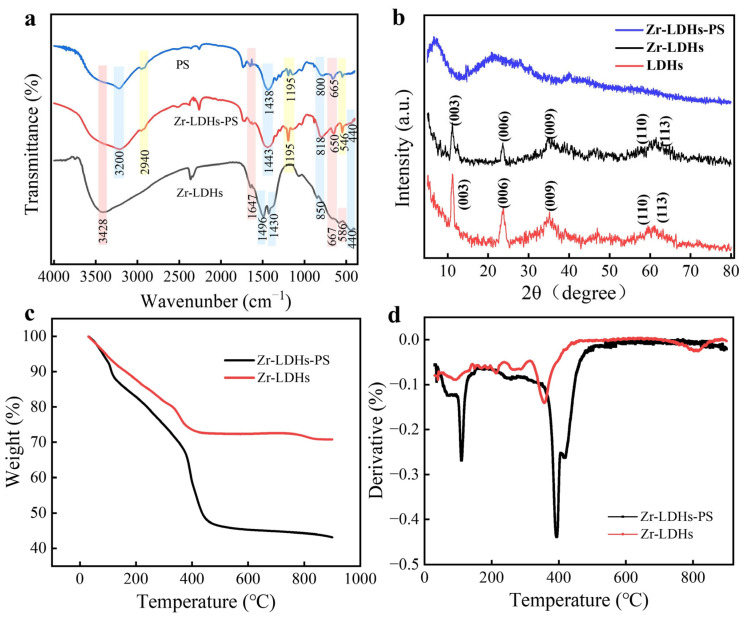
(**a**) FT-IR spectra and XRD patterns (**b**) of LDHs samples; (**c**) TG profiles and (**d**) Zr-LDHs-PS of Zr-LDHs and the Zr-LDHs-PS hydrogel.

To clarify the mechanisms behind the phosphate adsorption behavior of Zr-LDHs and the Zr-LDHs-PS hydrogel, structural characterization was performed. Figure 6a compares the FT-IR spectra of PS, Zr-LDHs, and the Zr-LDHs-PS hydrogel. A strong peak at 3420 cm^−1^ in Zr-LDHs relates to the O–H stretching vibration of interlayer water, characteristic of LDH materials [42]. Shoulder-like bands near 1430 and 1496 cm^−1^ result from the asymmetric stretching (ν_3_) of intercalated CO_3_^2−^ ions, a distinctive feature of carbonate-intercalated LDHs [43]. In contrast, the -OH bands of PS and the Zr-LDHs-PS hydrogel appear around 3220 cm^−1^. Pure PVA shows its -OH peak near 3280 cm^−1^, while that of SA is around 3300 cm^−1^ [44]. The shift in the -OH band in the composite indicates that repeated freeze–thaw cycles promote the formation of H-bonds and crystalline regions between PVA and SA, resulting in physical crosslinking which in turn reinforce the mechanical robustness of the PVA/SA hydrogel [45]. The band shape and width in the -OH region of the Zr-LDHs-PS hydrogel also encompass the characteristic 3420 cm^−1^ peak of Zr-LDHs, which is consistent with the composite nature of the material. Characteristic bands similar to those of LDHs were found in the FT-IR spectra of both Zr-LDHs-PS and Zr-PS hydrogels, confirming the presence of abundant hydroxyl groups favorable for anion exchange [46]. A band near 1640 cm^−1^ is attributed to the bending vibration of -OH from hydrogen bonding [47]. The absorption bands around 2940 and 1195 cm^−1^ in Zr-LDHs-PS correspond to the alginate biopolymer matrix, representing C-H stretching and C-O stretching vibrations, respectively, similar bands appear in the PS spectrum [48]. The band near 1450 cm^−1^ is attributed to CO_3_^2−^ bending, Zr^4+^ incorporation did not notably shift this peak. Bands between 850 and 440 cm^−1^ correspond to M-O stretching, confirming the presence of Mg^2+^, Fe^3+^, and Zr^4+^ ions [49]. The overlapping of these characteristic signals demonstrates the successful formation of the PVA/SA coated LDH composite.

#### 2.5.3. XRD

The XRD pattern of the LDH sample (Figure 6b) displayed characteristic diffraction peaks at 2θ = 11.3°, 22.5°, 34.1°, 59.5°, and 61.2°, which are assignable to the (003), (006), (009), (110), and (113) crystal planes, respectively. The (110) reflection reflects the in-plane structure, while the (113) reflection is associated with layer stacking [50]. Similar diffraction patterns were observed for Zr-LDHs. The incorporation of Zr^4+^ shifted the (003) peak to a lower angle, which is attributed to the increased net positive charge on the layers. This enhances electrostatic repulsion between layers, expands the interlayer spacing, and facilitates ion exchange [51]. Concurrently, the d-spacing of the (110) plane shifted toward higher angles, confirming successful Zr doping and the increased separation between metal cations due to the higher charge density [52]. In the Zr-LDHs-PS composite, a broad peak appeared at 2θ = 21.5° after encapsulation within the PS matrix. The hydrogel beads retained only weak diffraction features of the original Zr-LDHs powder, along with noticeable peak broadening and displacement [53].

#### 2.5.4. TG and DTG

To analyze the thermal decomposition profiles and stability of Zr-LDHs and the Zr-LDHs-PS hydrogel, thermogravimetric analysis was conducted. As shown in Figure 6c, Zr-LDHs exhibited three distinct mass-loss stages. The initial weight loss (30–300 °C, ~19%) corresponded to the removal of physically adsorbed and interlayer water, as indicated by small negative peaks in the DTG curve (Figure 6d). The main decomposition stage (300–450 °C, 9.1%) showed a strong DTG peak, attributed to dehydroxylation of the layers and decomposition of interlayer anions such as CO_3_^2−^. At higher temperatures (~800 °C, 1.7%), only minor residual decomposition occurred [54]. In contrast, Zr-LDHs-PS hydrogel displayed two major mass-loss stages. A pronounced initial loss (<150 °C, ~15%) resulted from the evaporation of bound water in the hydrophilic PVA/alginate matrix, along with the removal of water associated with the LDH component. The main decomposition stage (250–550 °C, ~33.7%) involved dehydration, chain scission, and carbonization of the polymers, overlapping with dehydroxylation and anion decomposition from the LDHs [55].

#### 2.5.5. XPS

**Figure 7:**
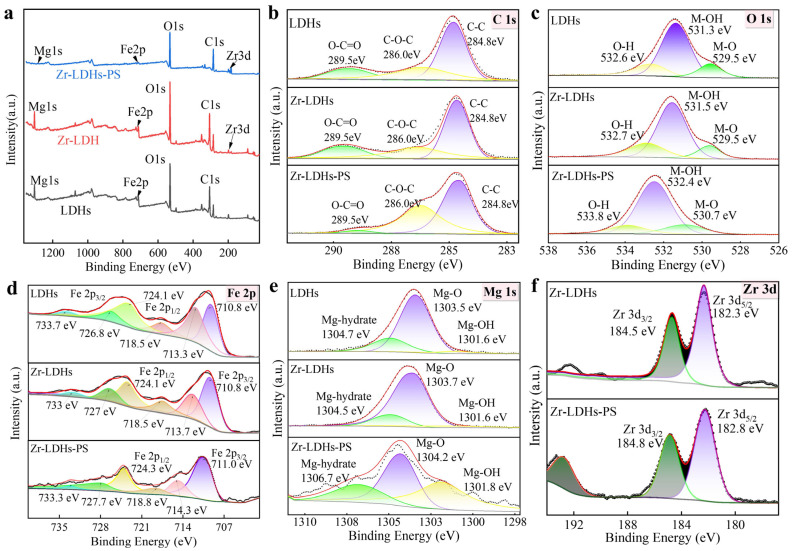
(**a**) Survey XPS spectra of the three materials; (**b**–**f**) C 1s, O 1s, Fe 2p, Mg 1s and Zr 3d high-resolution spectra.

The XPS spectra of LDHs, Zr-LDHs, and Zr-LDHs-PS gel are shown in Figure 7, including C 1s, O 1s, Mg 1s, Fe 2p, Zr 3d, and the full spectra. Further investigation was conducted via XPS analysis. The C 1s spectra of all three materials (Figure 7b) display three distinct peaks at 284.8, 286.1, and 289.0 eV, assigned to C-C, C-O-C, and C=O groups, respectively. Notably, a decrease in the peak area of the C=O group is observed. In Figure 7c, the O 1s peaks are deconvoluted into three components: O-H, M-O, and adsorbed M-OH (M = Zr, Mg, Fe). The proportion of the M-OH peak increases after coating the LDHs powder, indicating the presence of abundant hydroxyl groups in PVA-SA that are favorable for adsorption. In Figure 7d–f, Zr-LDHs powder is incorporated into the PVA-SA framework, the binding energies of Fe, Mg, and Zr shift to higher values, with shifts ranging from 0.2 to 2 eV. This unusual energy shift may be attributed to the extraction of electron density around phosphorus by the highly electronegative PVA-SA framework [56].

**Figure 8:**
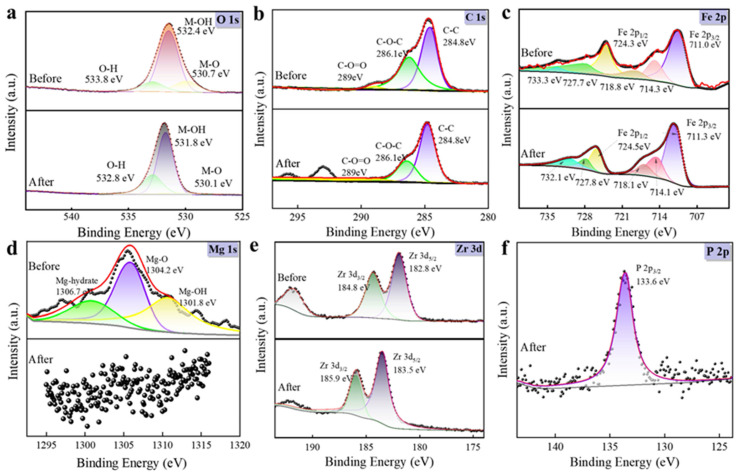
XPS spectra of Zr-LDHs-PS hydrogel before and after phosphate adsorption (**a**–**f**) O 1s, C 1s, Fe 2p, Mg 1s, P 2p and Zr 3d.

To elucidate the adsorption mechanism, XPS spectra of the Zr-LDHs-PS hydrogel were collected before and after phosphate exposure. The O 1s spectrum (Figure 8a) was fitted to three peaks arising from M-O, O-H, and adsorbed M-OH bonds. Notably, post-adsorption, the M-O peak contribution in Zr-LDHs-PS shows a significant reduction. This indicates that phosphate replaces hydroxyl groups via ligand exchange, forming M-O-P complexes through surface complexation [57]. The C 1s spectrum (Figure 8b) reveals contributions from C-C, C-O-C, and O-C=O groups, with binding energies of 284.8, 286.1, and 289.0 eV, respectively [58,59]. After phosphate adsorption, the Mg 1s and Fe 2p peaks shift slightly toward lower binding energies (Figure 8c,d). This points to an electron-withdrawing Lewis acid character of the adsorption sites, which remove electron density from the oxygen atoms of adsorbed phosphate [60]. Zr species and the LDH matrix are identified as the key contributors to phosphate adsorption, as evidenced by the experimental and characterization data. The Zr 3d spectrum before adsorption shows peaks at 186.1 eV and 183.8 eV (Figure 8e). After adsorption, these peaks shift to 185.9 eV and 183.5 eV, respectively, representing a decrease of 0.2–0.3 eV, it can be explained by electrons populate the Zr 4f orbital from the ligand valence band, indicating the formation of new Zr-O-P bonds [61,62]. As seen in Figure 8f, a P 2p_3/2_ peak appears at 133.6 eV, confirming successful binding of phosphate to the Zr-LDHs-PS hydrogel. Compared to the P 2p peak of pure KH_2_PO_4_ (~134.0 eV), a shift of 0.4 eV toward lower binding energy is observed. This demonstrates strong affinity between phosphate and the hydrogel, supporting that adsorption occurs via ion exchange, electrostatic attraction, or complexation [63].

#### 2.5.6. BET

**Figure 9:**
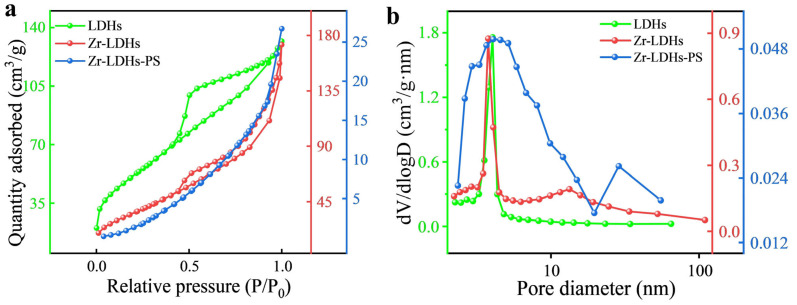
(**a**) N_2_ sorption isotherms and (**b**) pore size distribution profiles of LDHs, Zr-LDHs, and the Zr-LDHs-PS hydrogel.

To elucidate the textural properties of the adsorbents, LDHs, Zr-LDHs, and Zr-LDHs-PS were characterized by nitrogen adsorption–desorption measurements and pore size distribution analysis. Both powdered LDH and Zr-LDHs (Figure 9a) display type-IV isotherms with H_4_-type hysteresis, suggesting the coexistence of mesopores and slit-like pores from interlayer stacking [64]. The specific surface area of Zr-LDHs was 188.714 m^2^/g, compared to 135.591 m^2^/g for LDHs (Table 4). The introduction of Zr slightly disrupts the layered structure, leading to an increased surface area. The type-II isotherm and H_3_/H_4_ hysteresis observed for the composite microspheres (Figure 9b) are attributed to the PVA-SA phase, which creates irregular slit pores. Conversely, due to the absence of a stable framework, pure PVA-SA beads undergo severe shrinkage upon lyophilization, consequently yielding a very low surface area. In contrast, after incorporating Zr-LDHs, the composite microspheres attained a specific surface area of 26.116 m^2^/g.

### 2.6. Application Performance of Zr-LDHs-PS in Water, Soil and Plant

**Figure 10:**
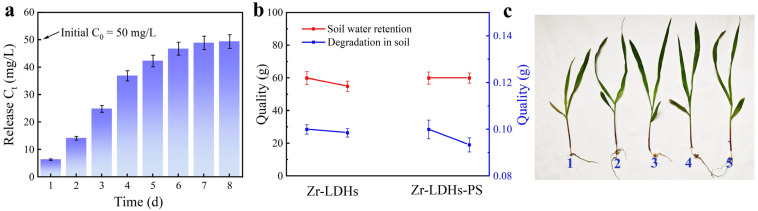
(**a**) Phosphate sustained-release results of Zr-LDHs-PS hydrogel; (**b**) soil water retention and self-degradation results of Zr-LDHs and Zr-LDHs-PS; (**c**) corn seedling cultivation with phosphate-adsorbed materials. Materials: (1) Control; (2) Commercial P fertilizer; (3) LDHs; (4) Zr-LDHs; (5) Zr-LDHs-PS.

The comprehensive performance of Zr-LDHs-PS hydrogel was evaluated through phosphate release, soil behavior, and plant growth experiments. As shown in Figure 10a, the hydrogel exhibited sustained phosphate release over 8 days in static deionized water (25 °C, pH = 6.5). The aqueous concentration improved from 6.4 mg/L (day 1) to 46.8 mg/L (day 6), stabilizing at 48.9–49.4 mg/L on days 7–8, indicating an initial release followed by equilibrium. Soil experiments (Figure 10b) demonstrated its effective water retention, with the Zr-LDHs-PS-treated system maintaining a stable mass of 60.01 g, significantly higher than the Zr-LDHs system (54.95 g). The composite also degraded more slowly (remaining mass: 0.096 g vs. 0.091 g), confirming enhanced soil stability [65]. In maize seedling tests (Figure 10c), the phosphate-loaded Zr-LDHs-PS treatment group (Group 5) demonstrated superior promotion of both root and shoot growth compared to commercial fertilizer. These results indicate that the synthesized Zr-LDHs-PS hydrogel material exhibits integrated properties of nutrient slow-release, water retention, and environmental stability, suggesting its potential applicability as an eco-friendly slow-release fertilizer [66].

### 2.7. Adsorption Mechanisms

**Figure 11:**
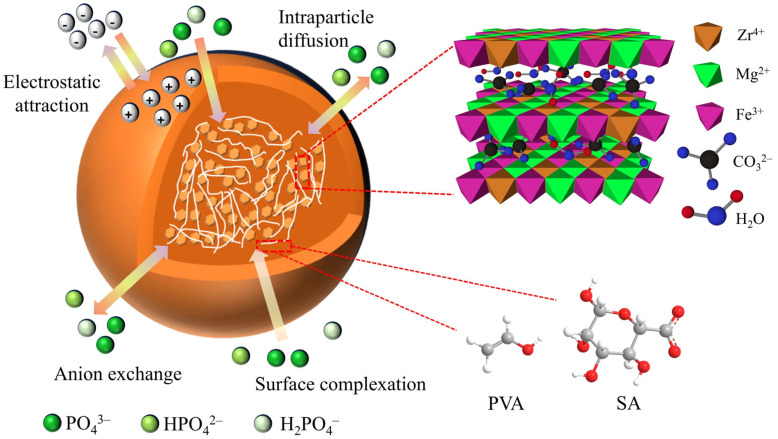
The possible adsorption mechanisms of Zr-LDHs-PS hydrogel for phosphate.

Possible mechanisms for phosphate adsorption by Zr-LDHs-PS in aqueous solution are illustrated in Figure 11. The multi-porous framework structure of the Zr-LDHs-PS hydrogel microspheres, as shown by SEM (Figure 5c), promotes the diffusion and immobilization of phosphate ions (PO_4_^3−^) inside the hydrogel matrix. During the synthesis of LDHs, the incorporation of Zr^4+^, Fe^3+^, and Mg^2+^ introduces abundant positive charges on the layers. Consequently, electrostatic attraction contributes to the primary adsorption stage of phosphate onto the Zr-LDHs loaded on PS. Kinetics modeling indicates the involvement of both diffusional transport and chemical adsorption (ligand exchange) in the adsorption process (Figure 2b). Furthermore, pH-dependent experiments (Figure 4a) reveal that when the pH outdo the pHpzc, electrostatic interaction should be suppressed. However, the efficiency of phosphate removal still increases to 93.4% at pH = 5. This result confirms the dominant role of a chemical adsorption mechanism. FT-IR spectra (Figure 6a) demonstrate the existence of abundant M–OH groups on the Zr-LDHs layers, which provide active sites for ligand exchange. After adsorption, the characteristic peak of M-OH shifts (Figure 7c), and a signal corresponding to P-O appears, indicating the exchange between phosphate and surface hydroxyl groups. XPS analysis offers further evidence: the Zr 3d peak shifts after adsorption (Figure 8e), suggesting the formation of a Zr–O-P coordination bond. A peak observed at 133.6 eV in the P 2p spectrum (Figure 8f), corresponding to P-O, confirms that an M-O-P complex formed through ligand exchange with LDH surface -OH groups. In summary, the exceptional phosphate adsorption performance of the Zr-LDHs-PS hydrogel microspheres is attributed to a synergistic process involving their porous structure, initial electrostatic attraction, and ultimately strong chemisorption via ligand exchange forming stable inner-sphere M-O-P complexes.

## 3. Conclusions

In this study, ZrMgFe-LDHs were immobilized into recoverable PVA/SA hydrogel beads, and the resulting Zr-LDHs-PS beads exhibited stable and efficient phosphate adsorption. The core hypothesis is that gel confinement enables nano-adsorbent recovery and phosphorus recycling, with the key innovation being the integration of chemisorption with slow-release fertilizer functionality to close the loop of “removal–recovery–agricultural use.” At 25 °C, the adsorption capacity reached 51.19 mg/g, following pseudo-second-order kinetics indicative of chemisorption. This exothermic chemisorption reduces capacity with increasing temperature per Le Chatelier’s principle, while elevated temperature also alters gel swelling, compresses diffusion channels, and impedes mass transfer. Compared with pristine LDH powder, the beads showed improved separability and stability, retaining >80.6% capacity after five cycles—outperforming most reported analogues. They also maintained high capacity across a wide pH range and tolerated competing anions (e.g., Cl^−^, NO_3_^−^), surpassing conventional LDH adsorbents. Mechanistic studies confirmed ion exchange, electrostatic interactions, and ligand exchange. Notably, the phosphate-loaded beads can be repurposed as a slow-release fertilizer-a feature absent in most literature focusing only on removal efficiency. Thus, this design links phosphate removal to nutrient recovery and circular utilization.

## 4. Materials and Methods

### 4.1. Materials

Details of the chemicals and their sources was provided in Text S1.

### 4.2. Preparation of Zr-LDHs

**Figure 12:**
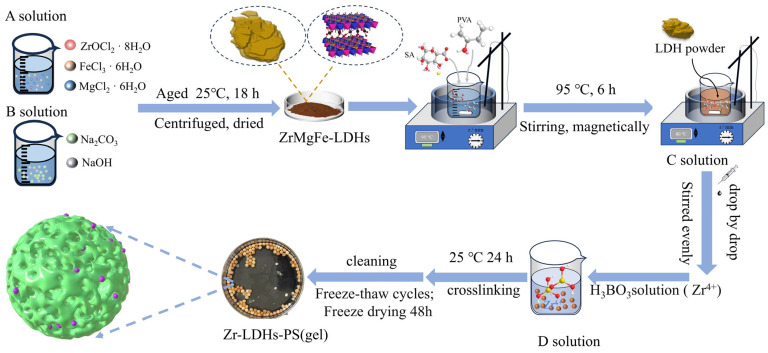
Synthesis procedure of Zr-LDHs-PS hydrogel microspheres.

Figure 12 illustrates the synthesis procedure of the LDHs. Based on a method reported in the literature [67], the LDHs were prepared using a controllable coprecipitation method. First, Solution A was prepared by dissolving 3.6929 g of MgCl_2_·6H_2_O, 3.5540 g of FeCl_3_·6H_2_O (Mg^2+^:Fe^3+^ molar ratio = 2:1), and 0.3719 g of ZrOCl_2_·8H_2_O in 15 mL of distilled water. Meanwhile, Solution B was obtained by dissolving 2.6620 g of Na_2_CO_3_ and 1.0000 g of NaOH were dissolved in another 15 mL of water. Both Solution A and Solution B were then slowly added dropwise into a beaker containing 10 mL of distilled water under constant stirring. During the addition, the pH of the mixture was maintained between 9 and 10. Following an 18 h aging period, the slurry was collected for further use. Subsequently, the precipitate was collected, centrifuged, and repeatedly washed with distilled water until the supernatant reached neutrality. The solid was then dried at 80 °C for 48 h, then ground and passed through a 100-mesh sieve, yielding a brown powder, denoted as Zr-LDHs. Other LDHs were prepared following a similar procedure.

### 4.3. Preparation of Zr-LDHs-PS

PS gel was synthesized via a dual-crosslinking method [68,69]. To achieve desired adsorption performance, Zr-LDHs were incorporated during the crosslinking process, and the ionic species in the boric acid solution were varied. First, 1.65 g PVA and 0.43 g SA were combined in 20 mL of deionized water. The mixture was magnetically stirred at 95 °C for 6 h to obtain a homogeneous PS solution. Subsequently, LDHs powder was incorporated into the solution. The mixture underwent stirring at 65 °C for 2 h and then was subjected to ultrasonication for 30 min to obtain uniformly dispersed suspensions. The concentrations of LDHs in the suspensions were 0%, 2%, 4%, 6%, and 8% (*W*/*V*), respectively. These suspensions were then dripped into a crosslinking solution using a peristaltic pump. The crosslinking solution contained saturated H_3_BO_3_ and one type of metal ion (Zn^2+^, Cu^2+^, Al^3+^, La^3+^, or Zr^4+^). The concentration of the metal ions was varied (0.05, 0.1, 0.15, 0.2, 0.25, or 0.3 mol/L). Upon contact with the crosslinking solution, the droplets rapidly gelled into beads and were soaked for 24 h. Afterward, the beads were rinsed repeatedly using deionized water until the pH of the washings fell within the range of 6 to 8. The beads were then undergoing two freeze–thaw cycles for further crosslinking. Each cycle involved freezing (−20 °C, 20 h) and thawing (room temperature 4 h). Finally, the product was freeze-dried for 48 h, stored in a sealed container for later use, and labeled as Zr-LDHs-PS.

### 4.4. Characterization of Adsorbents

The methodology for material characterization is provided in Text S2.

### 4.5. Batch Tests

Batch test processes were described in Text S3.

## Figures and Tables

**Table 1:** Parameters for modeling the phosphate adsorption process on the Zr-LDHs-PS hydrogel, including two kinetics models.

	Pseudo-First-Order Model			Pseudo-Second-Order Model		
C_0_ (mg/L)	q_e,cal_	k_1_	R^2^	q_e,cal_	k_2_	R^2^
25	5.601	0.087	0.995	5.821	0.045	0.998
50	11.654	0.088	0.999	11.981	0.028	0.999
75	16.198	0.064	0.995	17.275	0.008	0.999

**Table 2:** Parameters for isotherms modeling the phosphate adsorption process on the Zr-LDHs-PS hydrogel.

Isotherm	Langmuir			Freundlich		
Parameters	q_m_	K_L_	R^2^	K_f_	1/n	R^2^
15 °C	49.572	0.201	0.961	10.782	0.447	0.966
25 °C	51.313	0.203	0.982	11.006	0.455	0.979
35 °C	53.217	0.175	0.967	10.486	0.474	0.960

**Table 3:** Comparison of the adsorption capacity of phosphate between Zr-LDHs-PS and other adsorbents.

No	Adsorbent	Condition (pH)	Qe (mg/g)	Reference
1	Zn/Al/Zr LDH@HTCC	5	26.64	[13]
2	MgFe-Zr LDH	-	9.6	[19]
3	La/Fe–Cs composites	-	67.52	[31]
4	PVA-alginate hydrogel	5.0	11.5	[32]
5	MgAl-LDH-PVA/alginate Beads	-	1.543	[33]
6	Fe-Mg-Zr LDH beads	6.71	21.61	[18]
7	Zr@Alg Bent LDH beads	3	30.25	[34]
8	Zr-LDHs-PS hydrogel	5	51.31	This study

**Table 4:** Pore parameters of LDHs, Zr-LDHs and Zr-LDHs-PS hydrogels.

Samples	Specific Surface Area (m^2^/g)	Pore Volume (cm^3^/g)	Average Diameter (nm)
LDHs	135.591	0.226	6.678
Zr-LDHs	188.714	0.201	4.264
Zr-LDHs-PS	26.116	0.039	3.838

## Data Availability

The data supporting the findings of this study are available from the corresponding authors upon reasonable request.

## Supplementary Materials

The following supporting information can be downloaded at https://www.mdpi.com/article/10.3390/gels12070570/s1, Text S1: Materials and reagents; Text S2: Characterizations; Text S3: Batch tests; Figure S1: Pseudo-First-Order Model (a) and Pseudo-Second-Order Model (b) with linear equations for phosphate adsorption on Zr-LDHs-PS; Figure S2: Langmuir (a) and Freundlich (b) models with linear equations for phosphate adsorption on Zr-LDHs-PS; Figure S3: Zeta potential values of Zr-LDHs and Zr-LDHs-PS before and after adsorption. Figure S4: Photographs of Zr-LDHs-PS hydrogel beads (a) before and (b–e) after 48 h immersion at pH 3, 5, 7, and 9, showing maintained structural integrity across all tested pH conditions. Figure S5: Leaching concentrations of Mg^2+^, Fe^3+^, and Zr^4+^ from Zr-LDHs-PS hydrogel beads after 24 h immersion in deionized water at different pH values (3, 5, 7, 9).
