# Supplementary material for: A Dual-Functional Zr-Ion Crosslinked PVA-Alginate Hydrogel with Embedded ZrMgFe-LDH for Enhanced Phosphate Recovery
Source: Gels. 2026 Jun 28;12(7):570. doi: 10.3390/gels12070570 (PMC13409721; doi:10.3390/gels12070570)
Supplement: Supplementary file 1 [file gels-12-00570-s001.zip › gels-4364423-supplementary.pdf]

A Dual-Functional Zr-Ion Crosslinked PVA-Alginate Hydrogel with Embedded ZrMgFe-LDH for Enhanced Phosphate Recovery

Supplementary Information

Contents

Texts

Text S1 Materials

Text S2 Characterization of adsorbents

Text S3 Batch tests

Figures

Figure S1 Pseudo-First-Order Model (a) and Pseudo-Second-Order Model (b) with linear equations for phosphate adsorption on Zr-LDHs-PS.

Figure S2 Langmuir (a) and Freundlich (b) models with linear equations for phosphate adsorption on Zr-LDHs-PS

Figure S3. Zeta potential values of Zr-LDHs and Zr-LDHs-PS before and after adsorption.

Figure S3. Zeta potential values of Zr-LDHs and Zr-LDHs-PS before and after adsorption.

Figure S5 Leaching concentrations of Mg^{2+}, Fe^{3+}, and Zr^{4+} from Zr-LDHs-PS hydrogel beads after 24 h immersion in deionized water at different pH values (3, 5, 7, 9).

Text S1 Materials and reagents

$MgCl_2{\cdot}6H_2O$, $FeCl_3{\cdot}6H_2O$, and $ZrOCl_2{\cdot}8H_2O$ were supplied by Aladdin Biochemical Technology Co., Ltd. (Shanghai, China). KH_2PO_4 and H_3BO_3 were obtained from Macklin Biochemical Technology Co., Ltd. (Shanghai, China).

Na_2CO_3 and NaOH were provided by Sinopharm Chemical Reagent Co., Ltd. (Beijing, China). Sodium alginate (SA) and polyvinyl alcohol (PVA) were purchased from Sinopharm Chemical Reagent Co., Ltd. (Beijing, China). The layered double hydroxide powder was synthesized in our laboratory. All chemicals used in this study were of analytical grade or higher. Deionized water was prepared using a laboratory ultrapure water system.

Text S2 Characterizations

The crystal structure of the as-prepared samples was examined by XRD, Bruker D8 ADVANCE, Germany). The structural identification and analysis of the materials were performed using FTIR (Nicolet iS50, USA) within a wavenumber range of 400 to 4000 cm^{-1}. The surface elemental composition and functional groups were characterized by XPS (Thermo Fisher Escalab 250Xi, UK) with a scanning energy range of 0–1200 eV. Morphology and microstructure observations were conducted using SEM (Hitachi S-3400N, Japan) and TEM (JEM-2100F, JEOL, Japan). Elemental distribution was determined via EDX (FEI Quanta 400, Netherlands). The point of zero charge was measured to evaluate the surface charge properties. The specific surface area was determined from N_2 adsorption–desorption isotherms based on the BET (Quadrasorb SI analyzer, USA). Thermogravimetric and derivative thermogravimetric analysis (TGA/DTG) was performed on a SII Exstar 6300 instrument under a nitrogen atmosphere, with a heating rate of 10 °C/min from room temperature to 800 °C. EIS, CV and galvanostatic charge–discharge measurements were carried out on an electrochemical workstation (IviumStat, Netherlands). Detailed test conditions are described below. EPR (Bruker, EMXPLUS, Germany) was employed to identify the involved radicals, using 5,5-dimethyl-1-pyrroline-N-oxide (DMPO) and 2,2,6,6-tetramethyl-4-piperidone (TEMP) as spin-trapping agents. The toxicity of TC and its degradation intermediates was assessed using the T.E.S.T. software based on quantitative structure–activity relationship (QSAR) predictions.

Text S3 Batch tests

The phosphate adsorption performance of the hydrogel adsorbent was investigated through batch experiments. A phosphate stock solution with a concentration of 1000 mg P/L was prepared by dissolving 4.39 g of KH_2PO_4in deionized water. Solutions with the desired phosphate concentrations were obtained by diluting the stock solution. For each experiment, 100 mg of adsorbent was added to 25 mL of phosphate solution. The dispersions were oscillated at 298 K and 150 rpm in a water bath shaker for 2h except for the kinetic study.

Adsorption kinetic experiments were conducted using 100 mL phosphate solutions with initial concentrations of 25, 50, and 75 mg/L, respectively. Samples were taken periodically (0-180 min) from the reactor to examine the variation in phosphate concentration. For Zr-LDHs-PS, 0.1 g of adsorbent was added to 25 mL of solution, and the kinetic tests lasted for 3 h. The kinetic data were analyzed using two models (pseudo-first-order and pseudo-second-order). Isothermal adsorption experiments were performed with initial phosphate concentrations ranging from 5.0-200 mg/L, and the isotherm data were fitted using the Langmuir and Freundlich models. The initial pH of the phosphate solution was adjusted 1-9 using 0.1 mol/L HCl or NaOH to study its effect on phosphate removal. The competitive adsorption of common coexisting anions (CO_3^{2-}, Cl^-, NO_3^-, and SO_4^{2-}) was investigated. The influence of ionic strength was evaluated at 0.01, 0.05, and 0.1 mol/L. Experiments on pH and coexisting anion effects were carried out at a phosphate concentration of 50 mg/L.

After each experiment, the sample was taken from the reaction system and the liquid phase was separated by filtration through a 0.45 μm membrane. The phosphate concentration was determined by the molybdenum blue method and recorded as P element. A UV-vis spectrophotometer was used for the analysis. All experiments were performed in triplicate, and the average values were used for analysis. The phosphate removal efficiency (R) and the

adsorption capacity (mg/g) at time t (q_t) and at equilibrium (q_e) were calculated using the following equations:

$$\mathrm{R}(\%) = \frac{C_0 - C_e}{C_0} \times 100\% \tag{1}$$

$$q_e = \frac{(C_0\text{-}C_e)}{m} V \tag{2}$$

$$q_t = \frac{(C_0\text{-}C_t)}{m} V \tag{3}$$

where: C_0 is the initial phosphate concentration before adsorption; C_t and C_e are the concentration of phosphate at t min and equilibrium (mg/L), respectively; V is the volume of the phosphate solution (L); and m is the mass of the adsorbent used (g).

a

模型	BoxLucas1
方程	y = a*(1 - exp(-b*x))
绘图	C
a	5.60122 ± 0.07908
b	0.08727 ± 0.01405
Reduced Chi-Sqr	0.02305
R平方(COD)	0.99639
调整后R平方	0.99549

模型	BoxLucas1
方程	y = a*(1 - exp(-b*x))
绘图	B
a	11.65466 ± 0.02534
b	0.08847 ± 0.00225
Reduced Chi-Sqr	0.00237
R平方(COD)	0.99991
调整后R平方	0.99989

模型	BoxLucas1
方程	y = a*(1 - exp(-b*x))
绘图	D
a	16.19808 ± 0.24233
b	0.06441 ± 0.00714
Reduced Chi-Sqr	0.19545
R平方(COD)	0.99625
调整后R平方	0.99532

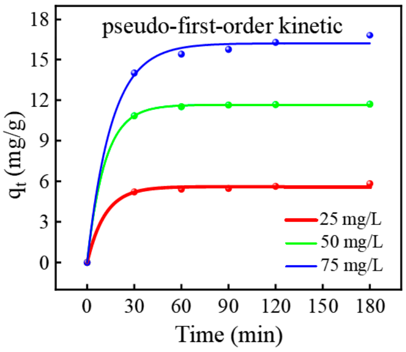

b

模型	dlx2 (User)
方程	y=((m^2)*n*x)/(1+m*n*x)
绘图	C
m	5.8217 ± 0.08345
n	0.045 ± 0.0114
Reduced Chi-Sqr	0.00867
R平方(COD)	0.99864
调整后R平方	0.9983

模型	dlx2 (User)
方程	y=((m^2)*n*x)/(1+m*n*x)
绘图	B
m	11.98197 ± 0.06572
n	0.02802 ± 0.00335
Reduced Chi-Sqr	0.00561
R平方(COD)	0.9998
调整后R平方	0.99975

模型	dlx2 (User)
方程	y=((m^2)*n*x)/(1+m*n*x)
绘图	D
m	17.27571 ± 0.16081
n	0.00804 ± 8.05606E-4
Reduced Chi-Sqr	0.02725
R平方(COD)	0.99948
调整后R平方	0.99935

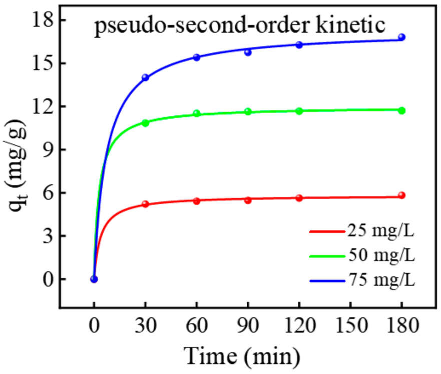

Figure S1. pseudo-first-order (a) and pseudo-second-order (b) models with linear equations for phosphate adsorption on Zr-LDHs-PS.

a

模型	LangmuirEXT1
方程	y = (a*b*x^(1-c))/(1 + b*x^(1-c))
绘图	Qe1
a	49.57158 ± 4.24549
b	0.20054 ± 0.05224
c	0 ± 0
Reduced Chi-Sqr	7.83635
R平方(COD)	0.96713
调整后R平方	0.96056

模型	LangmuirEXT1
方程	y = (a*b*x^(1-c))/(1 + b*x^(1-c))
绘图	Qe2
a	51.31283 ± 2.93782
b	0.20182 ± 0.03456
c	0 ± 0
Reduced Chi-Sqr	3.73598
R平方(COD)	0.9853
调整后R平方	0.98237

模型	LangmuirEXT1
方程	y = (a*b*x^(1-c))/(1 + b*x^(1-c))
绘图	Qe3
a	53.21749 ± 4.93438
b	0.1764 ± 0.04586
c	0 ± 0
Reduced Chi-Sqr	7.80429
R平方(COD)	0.96963
调整后R平方	0.96356

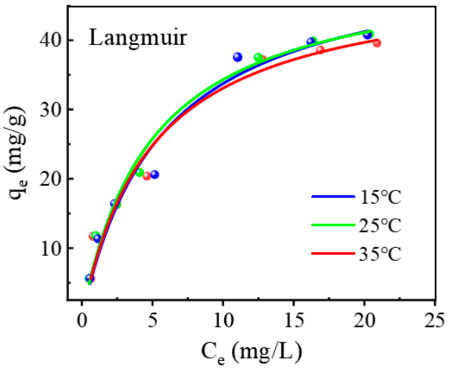

b

模型	Freundlich1 (User)
方程	y=a*x^(-b)
绘图	Qe1
a	10.78152 ± 1.30947
b	-0.44779 ± 0.0461
Reduced Chi-Sqr	6.63241
R平方(COD)	0.97218
调整后R平方	0.96662

模型	Freundlich1 (User)
方程	y=a*x^(-b)
绘图	Qe2
a	11.00627 ± 1.05173
b	-0.45498 ± 0.03658
Reduced Chi-Sqr	4.34993
R平方(COD)	0.98289
调整后R平方	0.97947

模型	Freundlich1 (User)
方程	y=a*x^(-b)
绘图	Qe3
a	10.4865 ± 1.46444
b	-0.47396 ± 0.05391
Reduced Chi-Sqr	8.60708
R平方(COD)	0.96623
调整后R平方	0.95948

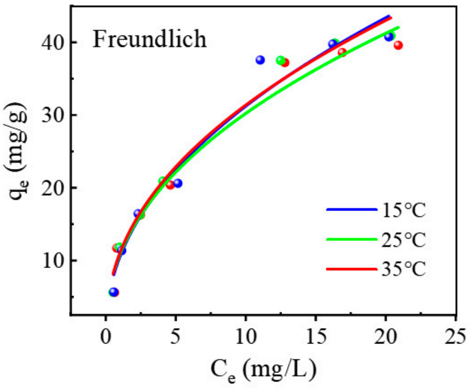

Figure S2. Langmuir (a) and Freundlich (b) models with linear equations for phosphate adsorption on Zr-LDHs-PS.

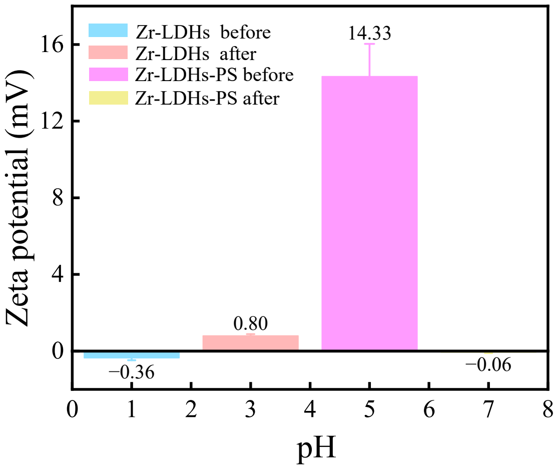

Figure S3. Zeta potential values of Zr-LDHs and Zr-LDHs-PS before and after adsorption.

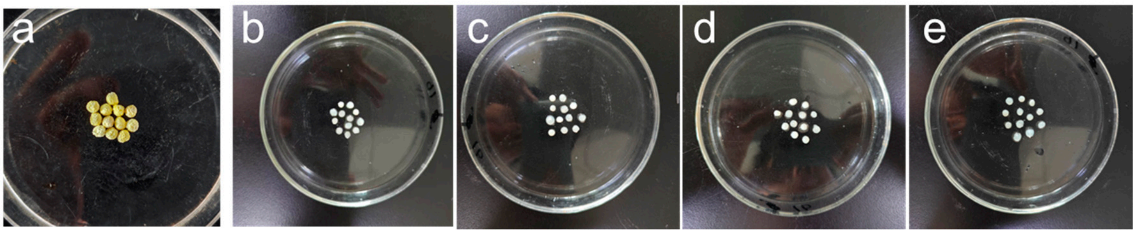

Figure S4. Photographs of Zr-LDHs-PS hydrogel beads (a) before and (b–e) after 48 h immersion at pH 3, 5, 7, and 9, showing maintained structural integrity across all tested pH conditions.

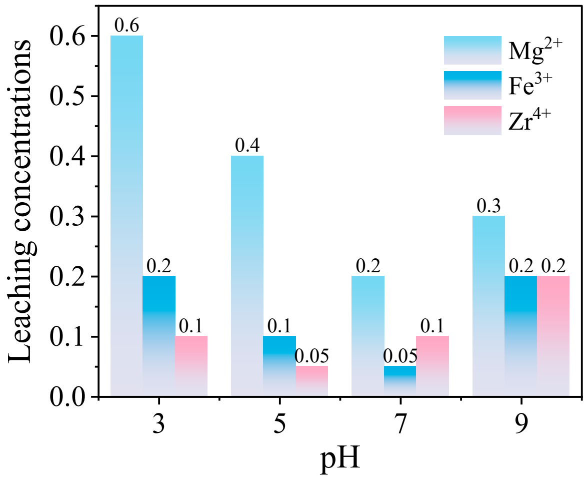

Figure S5. Leaching concentrations of Mg^{2+}, Fe^{3+}, and Zr^{4+} from Zr-LDHs-PS hydrogel beads after 24 h immersion in deionized water at different pH values (3, 5, 7, 9).
